# Separate BNST Microcircuits Targeted by Direct Versus Amygdala-Relayed Prefrontal Inputs Mediate Dissociable Phenotypes After Isolation

**DOI:** 10.3390/cells15020116

**Published:** 2026-01-08

**Authors:** Hongxia Yuan, Yongmei Zhong, Xuehan Zhang

**Affiliations:** State Key Laboratory of Medical Neurobiology, MOE Frontiers Center for Brain Science, Institutes of Brain Science, Fudan University, Shanghai 200032, China; hxyuan19@fudan.edu.cn (H.Y.);

**Keywords:** anxiety-like behavior, depression-like behavior, social motivation, social recognition, prefrontal cortex, basolateral amygdala, bed nucleus of the stria terminalis

## Abstract

Anxiety, depression, and social impairment exhibit high clinical comorbidity, yet their underlying shared neural circuitry remains poorly defined. Using a mouse model of chronic social isolation combined with circuit tracing and chemogenetic tools, we identified a key role for the basolateral amygdala (BLA) in relaying prefrontal cortex (PFC) signals to the bed nucleus of the stria terminalis (BNST) to drive behavioral changes. Further circuit dissection identified two distinct BNST microcircuits segregated by their input sources: one receives indirect PFC input relayed through the BLA (PFC → BLA → BNST), while the other is innervated by direct PFC projections (PFC → BNST). Chemogenetic inhibition of BLA neurons in the indirect pathway ameliorated anxiety-like behavior, depression-like behavior, and social deficits. Within the BNST, however, inhibition of neurons in PFC → BLA → BNST pathway selectively alleviated affective phenotypes without altering social behavior. In contrast, inhibition of neurons in PFC → BNST pathway specifically restored social recognition while leaving emotional behaviors intact. Thus, the BLA integrates PFC-derived signals to broadly modulate behavior, while downstream BNST microcircuits dissociate these influences. The indirect, BLA-relayed pathway within the BNST specifically drives affective symptoms, whereas the direct PFC → BNST pathway selectively governs social recognition. This dissociable circuit model offers a new framework for understanding clinical comorbidity and may inform targeted interventions for distinct symptom dimensions.

## 1. Introduction

Anxiety, depression, and social dysfunction are highly comorbid clinically, representing a significant public health challenge [1]. Epidemiological data confirm that their co-occurrence far exceeds chance, with most patients experiencing concurrent affective and social impairments—a cycle that often leads to treatment resistance and poor prognosis [2,3,4].

Neuroimaging studies consistently show reduced prefrontal cortex (PFC) activity and dysregulated control over limbic regions such as the amygdala and bed nucleus of the stria terminalis (BNST) in these patients [5,6,7,8,9]. Yet, interventions targeting these broad circuits yield inconsistent outcomes [10], suggesting that functionally specific microcircuits within these networks may separately regulate anxiety, depression, and social behavior [11]. Thus, dissecting the organization of these parallel pathways at the circuit level is essential for unraveling the neural basis of comorbidity.

The PFC, as a central region for cognitive control and stress regulation, is essential for both emotional and social behavior [12,13]. Chronic stress can induce significant maladaptive plasticity in the PFC, including dendritic spine loss and impaired glutamatergic synaptic transmission, ultimately leading to a “hypofrontality” state [14,15]. This weakened top-down control capacity promotes anxiety-like behaviors and impairs social preference [16]. Importantly, the functional output of the PFC is markedly pathway-specific. For example, projections from the ventromedial PFC to the nucleus accumbens primarily regulate motivation and reward-related behavior [17], while inputs from the prelimbic cortex to the amygdala play a key role in fear extinction and social interaction [18,19,20]. These findings suggest that the PFC may regulate different behavioral dimensions through parallel, functionally segregated downstream pathways [21].

The basolateral amygdala (BLA) serves as a hub for emotional integration, receiving convergent inputs from sensory, thalamic, and prefrontal regions [22,23]. Under stress conditions, BLA hyperactivity is associated with anxiety-like behaviors [24,25], whereas its inhibition disrupts social recognition [26]. BLA neurons display projection-defined functional specialization: outputs to the ventral hippocampus exert anxiolytic and pro-social effects [27], while those to the central amygdala primarily drive fear responses [28]. This projection-dependent functional organization establishes the BLA as a critical node linking cognitive processing and emotional expression [29,30,31]. Building on this evidence, we propose that the BLA acts as a pivotal interface downstream of the PFC, channeling information into distinct parallel pathways that contribute to the regulation of comorbid behavioral symptoms.

The BNST, a core component of the extended amygdala, plays a pivotal role in sustaining anxiety and integrating stress responses [32,33]. It receives dual inputs from both the PFC and the basolateral amygdala [34,35], and its intrinsic neurons exhibit high heterogeneity [36,37]. At the circuit level, optogenetic studies show that activating the PFC → BNST pathway induces long-lasting anxiolytic effects [38], while activating specific BLA → BNST pathways promotes anxiety [39,40,41]. Concurrently, specific neuronal populations within the BNST (such as AVP receptor-positive or OXT receptor-positive cells) have been confirmed to participate in the regulation of social behavior [42,43,44]. These lines of evidence collectively establish the BNST as a key hub for the convergence of cognitive and emotional information [33]. However, a fundamental question remains unresolved: Under pathological conditions, are the two major input pathways converging onto the BNST—the direct pathway originating from the PFC and the indirect pathway relayed via the BLA—functionally independent? Do they specifically regulate emotional states (anxiety/depression) and social behavior, respectively, by engaging distinct microcircuits within the BNST?

Based on the high clinical comorbidity of anxiety, depression, and social impairment and their potential shared neural mechanisms, this study proposes a core hypothesis: under stress conditions, emotional states and social behavior are regulated by two functionally distinct neuronal ensembles within the BNST—one that receives indirect PFC input relayed via the basolateral amygdala and mediates affective responses, and another that receives direct PFC input and specifically controls social behavior. To test this hypothesis, we employed a chronic social isolation stress mouse model and systematically investigated the functional properties of these two BNST neuronal ensembles by integrating neural circuit tracing, chemogenetic manipulation, and behavioral analysis. Experimental results demonstrated that (1) inhibiting basolateral amygdala neurons within the PFC → BLA → BNST pathway simultaneously improved anxiety-like and depression-like behaviors and alleviated social deficits; (2) specifically inhibiting the BNST neurons in the PFC → BLA → BNST pathway alleviated emotional symptoms without affecting social behavior; and (3) selectively inhibiting the BNST ensemble receiving direct PFC input specifically reversed social impairment without altering emotion-related behaviors.

This study demonstrates for the first time at the circuit level that BNST regulates stress-induced emotional and social impairments through two parallel microcircuits within its structure. These two microcircuits are distinguished by their input sources: one integrates indirect PFC input relayed through the BLA, while the other receives direct PFC input. Despite the BLA’s broad influence on behavior, functionally distinct subpopulationsin the BNST functionally segregate these effects: neurons receiving BLA-relayed PFC input specifically mediate affective symptoms, whereas those receiving direct PFC projections selectively govern social recognition. This discovery not only provides an innovative “input-defined functional dissociation” circuit model—highlighting the essential relay role of the BLA—to explain clinical comorbidity but, more importantly, establishes a theoretical foundation for developing precise interventions targeting specific symptom dimensions. It further points to potential neuromodulation strategies focused on distinct neuronal ensembles within the BNST.

## 2. Materials and Methods

Male C57BL/6J wild-type (C57) mice (3-week-old) were sourced from the Shanghai Slack Laboratory Animal Center. Mice were maintained in a temperature-controlled environment (22 ± 2 °C) under a 12 h light/dark cycle (lights on: 7:00 a.m.–7:00 p.m.), with ad libitum access to food and water. All experimental protocols received approval from the Animal Ethics Committee of the School of Basic Medical Sciences, Fudan University (Shanghai, China) and were performed in compliance with the National Institutes of Health Guide for the Care and Use of Laboratory Animals (1996). Measures were taken to minimize the number of animals utilized and to alleviate any potential suffering.

### 2.1. Social Isolation

Male C57 mice were individually housed from 3 to 7 weeks of age, followed by a series of behavioral tests. Group-housed mice were housed 3–5 per cage.

### 2.2. Behavioral Assessment

*Open field test*

Anxiety-like behavior was assessed using the open field test. Mice were individually placed in the center of a square arena (40 × 40 × 40 cm) and allowed to explore freely for 10 min. The test was recorded by an overhead camera, and the following parameters were analyzed using EthoVision XT 14 software (Noldus Information Technology, Wageningen, Gelderland, The Netherlands) to evaluate anxiety-like behavior: time spent in the central zone (20 × 20 cm), number of central entries, percentage of center distance travelled, thigmotaxis index, and total distance travelled.

*Elevated plus maze*

Anxiety-like behavior was further evaluated using the elevated plus maze. The apparatus consisted of two open arms and two enclosed arms (each 30 × 6 cm; enclosed arms with 15 cm-high walls) extending from a central platform (6 × 6 cm), elevated 50 cm above the floor. Mice were placed on the center platform facing an open arm and allowed to explore for 6 min. Data from the first 5 min were analyzed with EthoVision XT 14 software (Noldus, Noldus Information Technology, Wageningen, Gelderland, The Netherlands) for the following measures: time spent in the open and enclosed arms, number of entries into each arm type, percentage of distance travelled in each arm, head-dipping frequency, latency to first enter a closed arm, and total distance travelled.

*Social Interaction Test*

The social interaction test was performed to assess social motivation, following a previously established protocol [45]. The apparatus consisted of a white plexiglas box divided into three identical compartments (each 20 cm L × 48 cm W × 40 cm H) by transparent partitions with rectangular openings (10 cm wide) equipped with sliding doors to allow free movement between chambers. Twenty-four hours before testing, subject mice were habituated to the empty apparatus for 5 min. On the test day, after at least 1 h of acclimation to the testing room, an unfamiliar same-sex mouse (novel mouse) and an inanimate object were enclosed in wire cages and placed in the left and right chambers, with their positions randomized across trials. The test mouse was placed in the center chamber for 5 min with the sliding doors closed. The doors were then opened, allowing the test mouse to freely explore all three chambers for 10 min. The time spent in close proximity (within a 3–5 cm zone) to either the novel mouse or the object was recorded. Additionally, the total time spent within each chamber of the apparatus was recorded via an overhead infrared camera.

*Social Recognition Test*

The social recognition test was conducted immediately after the social interaction test to evaluate the ability to discriminate a novel from a familiar conspecific. The test mouse was temporarily removed from the apparatus. The inanimate object was then replaced with a second unfamiliar mouse (novel mouse, S2), while the original novel mouse remained in its chamber and was designated the “familiar mouse” (S1). The test mouse was then allowed to freely explore all three chambers for 10 min. The time spent in close proximity (within a 3–5 cm zone) to either the familiar mouse (S1) or the novel mouse (S2) was recorded. Additionally, the time spent in the chamber containing each mouse was recorded via an overhead infrared camera.

*Tail Suspension Test (TST)*

The tail suspension test (TST) was employed to evaluate depression-like behavior in mice. Briefly, each mouse was securely suspended by the tail using adhesive tape attached to the midpoint of the tail, approximately 2 cm from the tip. The animal’s behavior was recorded for a 6 min session. The immobility time of the last 4 min was recorded and analyzed. All experiments were conducted under standardized lighting and noise conditions.

*Forced Swimming Test (FST)*

The forced swimming test (FST) was employed to evaluate depression-like behavior in mice. Each animal was individually placed in a glass cylinder (16 cm diameter, 32 cm height) filled with water (25 ± 1 °C) to a depth of 20 cm for a 6 min test session. The immobility time of the last 4 min was recorded and analyzed. A longer immobility time was interpreted as increased depression-like behavior.

### 2.3. Virus Injection

Mice were anesthetized with isoflurane and fixed in a stereotaxic frame (RWD Life Science Co., Ltd., Shenzhen, Guangdong, China) with the skull leveled. Following a midline scalp incision, AAV was injected into the BLA, PFC, and BNST using a Nanoliter microinjector (World Precision Instruments, Sarasota, FL, USA). A total volume of 200 nL was administered at each site at a flow rate of 20 nL/min. The injection needle was retained for 10 min post-infusion to facilitate viral diffusion and minimize reflux. After wound closure, mice were housed for a 4-week recovery and viral expression period before any further experiments. All coordinates are given relative to bregma: BLA (AP −1.2 mm, ML ±3.0 mm, DV −4.9 mm), PFC (AP +2.0 mm, ML ±0.25 mm, DV −2.0 mm), and BNST (AP: +0.14 mm, ML: ±0.9 mm, DV: −4 mm). Detailed information regarding the viruses used in this study—including the vendor, catalog number, concentration, and storage conditions—is provided in Appendix A, Table A1.

The AAVs injections used in this study were as follows in Table 1:

### 2.4. Chemogenetic Manipulation

Clozapine N-oxide (CNO, MCE, HY-17366) was dissolved in 0.9% saline for intraperitoneal (i.p.) injection. To chemogenetically inhibit the targeted neuronal populations, CNO (1 mg/kg) was administered 30 min prior to each behavioral test. This regimen was designed to suppress the activity of: (1) BLA relay neurons that receive input from the PFC and project to the BNST. (2) BNST neurons in the PFC → BLA → BNST pathway; and (3) BNST neurons receiving direct projections from the PFC. The vendor, catalog number, concentration, and storage information for CNO (Clozapine N-oxide) are provided in Appendix A, Table A1.

### 2.5. Immunohistochemistry

Mice were deeply anesthetized with isoflurane and administered sodium pentobarbital (0.1 mL/kg, i.p.), followed by transcardial perfusion with 0.9% saline and ice-cold 4% paraformaldehyde (PFA). Brains were post-fixed overnight in the same fixative at 4 °C, cryoprotected in a graded sucrose series (10%, 20%, and 30% in PB), and sectioned coronally at 35 μm on a cryostat (Leica CM1950, Nussloch, Wetzlar, Germany). Free-floating sections were post-fixed in 4% PFA for 20 min, rinsed in PBS, and blocked with 5% donkey serum containing 0.1% Triton X-100 in PBS for 1.5 h at room temperature. Sections were then incubated overnight at 4 °C with rabbit anti-c-Fos antibody (1:1000, Abcam ab190289), followed by incubation with Alexa Fluor 488-conjugated donkey anti-rabbit secondary antibody (1:500, Abcam A21206) for 2 h at room temperature. After final washes, sections were mounted with fluorescent mounting medium.

### 2.6. Quantification of c-Fos Immunostaining

The group-housing mice and the isolation-housing mice were anesthetized and perfused, and c-Fos immunohistochemistry staining was performed. Tissue sections were imaged using an Olympus VS120 slide scanner (Olympus Corporation, Tokyo, Japan). For each brain region and animal, three sections were analyzed. Quantification was performed using ImageJ 1.x software (National Institutes of Health, Bethesda, MD, USA).

### 2.7. Statistical Analysis

Data are expressed as mean ± SEM. Normality and homogeneity of variances were assessed using the Shapiro–Wilk tests and Levene’s tests, respectively. For two-group comparisons, normally distributed data with equal variances were analyzed using a two-tailed Student’s *t*-test; data that were normally distributed but had unequal variances were analyzed using Welch’s *t*-test. Non-normally distributed data were analyzed using the Mann–Whitney U test. For the analysis of social preference or the social novelty preference, the time spent close with a novel mouse versus a familiar mouse (or an inanimate object) within the same trial was compared using two-tailed paired-samples *t*-tests. Significance levels are indicated as follows: *** *p* < 0.001; ** *p* < 0.01; * *p* < 0.05, ns: no significance. All statistical analyses were conducted using SPSS 27 (International Business Machines Corporation, Armonk, NY, USA).

## 3. Results

To establish a comorbid model of anxiety-depression and social behavior, this study employed an adolescent social isolation mouse model. Extensive evidence indicates that chronic social isolation reliably induces multiple behavioral abnormalities: for instance, it can elicit anxiety-like behaviors [46,47], depression-like phenotypes [48,49], as well as deficits in social and recognition capabilities [50,51]. Based on this, three-week-old male C57BL/6J mice were randomly assigned to two groups: the isolation group was housed individually, while the control group was group-housed in standard cages (3–5 mice per cage).

### 3.1. Social Isolation Induces a Complex Behavioral Syndrome and Co-Activation of Specific Brain Regions

**Figure 1:**
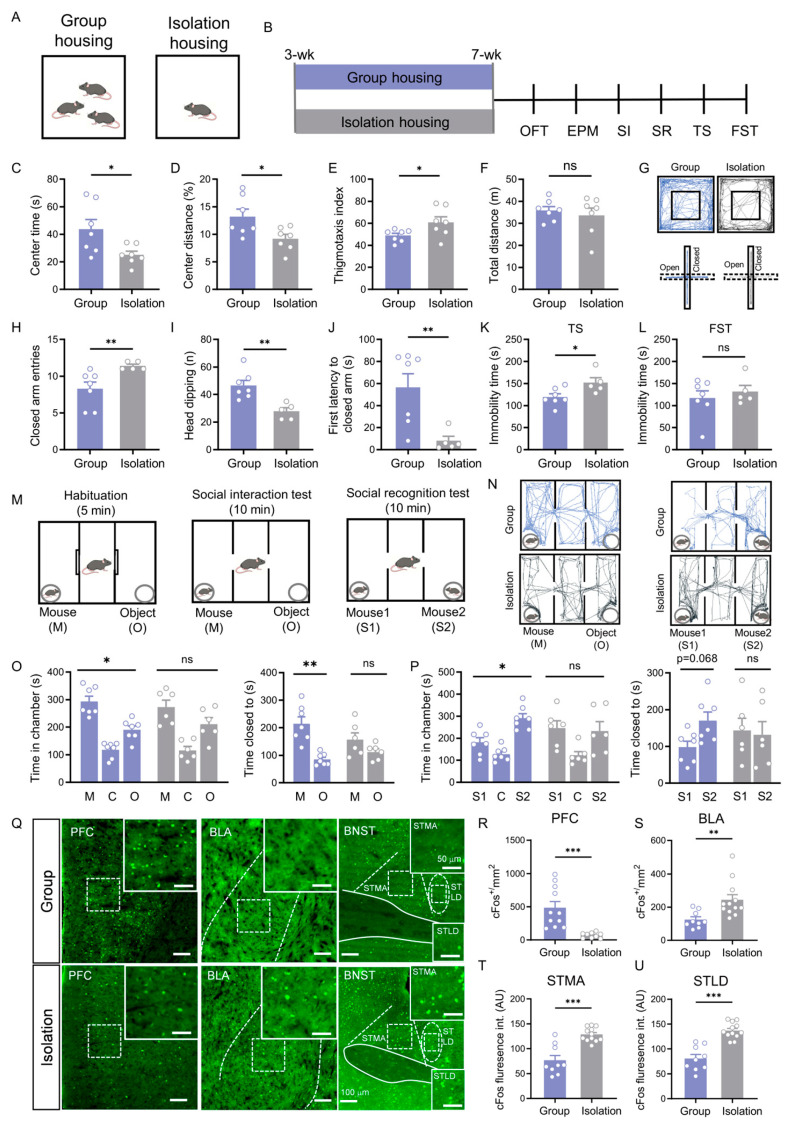
Social isolation induces affective deficits and impairs social motivation and recognition, associated with neural changes in the PFC, BLA, and BNST. (**A**) Schematic of the experimental housing paradigm (group housing vs. isolation housing). (**B**) Experimental timeline. (**C**–**F**) Open field test results: center time ((**C**), *p* = 0.038, Welch’s *t* test), center distance percentage ((**D**), *p* = 0.025, Student’s *t* test), thigmotaxis index ((**E**), *p* = 0.048, Student’s *t* test), and total distance traveled ((**F**), *p* = 0.565, Student’s *t* test) in group-housed (*n* = 7) and isolation-housed (*n* = 7) mice. (**G**) Representative movement trajectories in the open field (**upper**) and elevated plus maze (**lower**) tests. (**H**–**J**) Elevated plus maze results for group-housed (*n =* 7) and isolation-housed (*n =* 5) mice: closed arm entries ((**H**), *p* = 0.005, Mann–Whitney U test), head-dipping counts ((**I**), *p* = 0.003, Student’s *t* test), and latency to closed arms ((**J**), *p* = 0.005, Mann–Whitney U test). (**K**,**L**) Immobility time in tail suspension ((**K**), *p* = 0.03, Student’s *t* test) and forced swimming (**L**) tests (*p* > 0.05, Student’s *t* test). (**M**–**P**) Social behavior tests: (**M**) Schematics of social interaction (**left**) and social recognition (**right**) tests; On the day of testing, mice were first habituated in the three-chamber apparatus for 5 min, followed by a 10 min social interaction test and then a 10 min social recognition test.; (**N**) Representative trajectories in social interaction tests (**left**) and social recognition test (**right**); (**O**) Social interaction test results (time in chambers: mouse vs. object–Group *p* = 0.019, Isolation *p* = 0.236; proximity time: mouse vs. object–Group *p* = 0.007, Isolation *p* = 0.244; paired *t* test); (**P**) Social recognition test results (time in chambers: S1 vs. S2–Group *p* = 0.03, Isolation *p* = 0.869; proximity time: S1 vs. S2–Group *p* = 0.068, Isolation *p* = 0.860; paired *t* test). (**Q**) Representative c-Fos immunohistochemistry images in the PFC, BLA, and BNST of group-housed (**upper**) and isolation-housed (**lower**) mice. Scale bar: 100 μm. Boxed areas indicate corresponding magnified regions. Scale bar: 50 μm. (**R**,**S**) Quantitative analysis of c-Fos–positive neuron density in the PFC (R, *p* < 0.001, Student’s *t* test) and BLA ((**S**), *p* = 0.002, Mann–Whitney U test). (**T**,**U**) Quantitative analysis of c-Fos immunofluorescence intensity in the STMA (**T**) and STLD (**U**) (*p* < 0.001, Student’s *t* test). All data: mean ± SEM; * *p* < 0.05, ** *p* < 0.01, *** *p* < 0.001; ns = not significant.

In the open field test, isolation-housing (IH) mice exhibited significant reductions in the time spent in the center zone, the percentage of distance traveled in the center zone, and the thigmotaxis index (*p* < 0.05), without a change in total locomotion (*p* = 0.565; Figure 1C–F). This anxiety-like behavior was further corroborated in the elevated plus maze, where IH mice displayed more closed arm entries, fewer risk-assessment head dips, and a significantly shorter latency to first enter a closed arm (all *p* < 0.01; Figure 1H–K).

Furthermore, the behavioral deficits in isolated mice encompassed a depressive-like state. IH mice exhibited a significant increase in immobility time during the last four minutes of the tail suspension test (*p* < 0.05; Figure 1K) but not in the forced swim test (Figure 1L), indicating that social isolation also induced depression-like behavior.

In the social interaction test, group-housing (GH) control mice spent significantly more time in the chamber with a stranger mouse (*p* = 0.019) and more time in close proximity to it compared to the object (*p* = 0.007) (Figure 1O). In contrast, IH mice showed no such preference in either measure (chamber time: *p* = 0.236; proximity time: *p* = 0.244) (Figure 1O). During the social recognition phase, GH controls spent more time in the chamber with a novel mouse (S2) (*p* = 0.030) and in close proximity to it over the familiar one (S1) (*p* = 0.068) (Figure 1P). IH mice, however, displayed no preference in the social recognition test (chamber time: *p* = 0.869; proximity time: *p* = 0.860) (Figure 1P). Based on the above findings, these results indicate that social isolation not only induces anxiety-like and depression-like behaviors but also leads to significant social behavioral deficits.

Given the profound behavioral deficits, we asked whether neuronal activity in key brain regions implicated in social isolation was altered. Using c-Fos immunostaining as a marker of neuronal activation, we quantified the number of c-Fos-positive cells in PFC, BLA and BNST. c-Fos immunostaining revealed that social isolation led to opposing patterns of neuronal activation: the density of c-Fos-positive neurons in the PFC was significantly reduced (*p* < 0.001, Figure 1R), whereas in the BLA it was markedly increased (*p* < 0.01, Figure 1S). Furthermore, the fluorescence intensity of c-Fos-positive neurons in both the bed nucleus of the stria terminalis, medial division, anterior part (STMA) and the bed nucleus of the stria terminalis, lateral division, dorsal part (STLD) was significantly enhanced (all *p* < 0.001; Figure 1T,U). These region-specific changes in neural activity are linked to the observed behavioral syndrome.

While extant studies have firmly established the roles of the PFC → BLA [25], PFC → BNST [38], and BLA → BNST projections [39,40,41,52] in regulating affective behaviors, the overall architecture of this tripartite circuit remains fragmented. It is unknown whether these projections constitute parallel, independent pathways or are organized into an integrated hierarchy, such as a serial PFC → BLA → BNST circuit. To resolve this critical gap and to provide a structural framework for understanding their co-activation in social isolation (Figure 1), we systematically mapped the synaptic connectivity between the PFC, BLA, and BNST.

### 3.2. Anatomical Dissection of Parallel Prefrontal Pathways to the BNST

**Figure 2:**
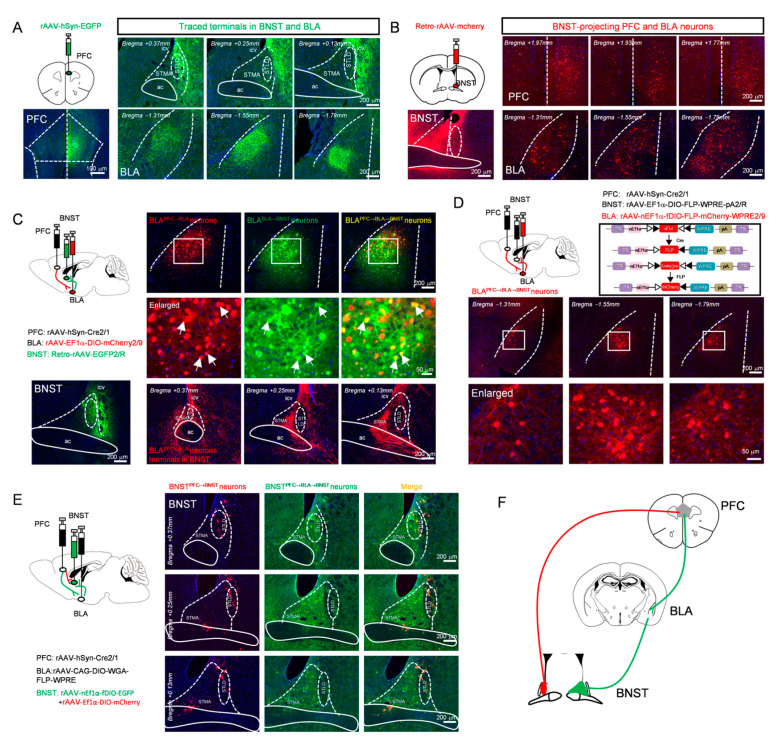
Two distinct populations of BNST neurons receive direct (BNST^PFC → BNST^ neurons) versus indirect PFC inputs (BNST^PFC → BLA → BNST^ neurons). (**A**) Anterograde viral injection into the PFC, schematic (**top left**) and expression at the injection site (**bottom left**, scale bar: 100 μm), showing representative images of terminal distribution in the BNST (**top**) and BLA (**bottom**). (**B**) Retrograde viral injection into the BNST, schematic (**top left**) and expression at the injection site (**bottom left**), showing representative images of retrogradely labeled neurons in the PFC (**top**) and BLA (**bottom**). (**C**) Schematic of Cre virus injection into the PFC combined with Cre-dependent DIO virus injection into the BLA and retrograde labeling from the BNST (**top left**) and BNST injection site (**bottom left**), showing labeled PFC-projected BLA neurons (**top**), magnified view (**middle**, arrows denote BLA neurons receiving PFC afferents and projecting to the BNST), and terminal distribution of PFC → BLA axons within the BNST (**bottom**). (**D**) Labeling BLA relay neurons in PFC → BLA → BNST pathway: schematic (**top left**) and viral strategy (**top right**), showing distribution (second row) and magnified view (third row) of labeled neurons. (**E**) Labeling BNST^PFC → BLA → BNST^ neurons and BNST^PFC → BNST^ neurons, representative expression images (first column: BNST^PFC → BNST^ neurons (red); second column: BNST^PFC → BLA → BNST^ neurons (green); third column: merged) (**F**) Schematic diagram of brain region projection relationships. All scale bars: whole images, 200 µm; magnified insets, 50 µm.

To clarify the connectivity between the PFC, BLA, and BNST, we performed a series of viral tracing experiments. First, we injected an anterograde adeno-associated virus (AAV) expressing EGFP into the PFC, which revealed robust axonal terminals projecting from the PFC to both the BLA and the BNST (Figure 2A). To verify that the PFC and BLA are direct upstream inputs to the BNST, we injected a retrograde AAV expressing mCherry into the BNST. This approach successfully labeled neuronal populations in both the PFC and BLA (Figure 2B). Together, these results demonstrate that the BNST receives direct inputs from both the PFC and BLA, anatomically defining the potential indirect pathway PFC → BLA → BNST alongside the parallel direct pathway PFC → BNST.

To further verify the functional viability of this potential indirect pathway, a dual-virus labeling strategy was employed. Specifically, an anterograde trans-synaptic adeno-associated virus (rAAV 2/1-Cre) was injected into the PFC, a Cre-dependent reporter virus (AAV2/9-EF1α-DIO-mCherry) into the BLA, and a retrograde green fluorescent tracer into the BNST. Doubly labeled neurons (yellow) were observed in the BLA, indicating the presence of BLA “relay neurons” that receive input from the PFC and project to the BNST. Examination of the BNST confirmed that axonal terminals from these BLA neurons were densely distributed in the STMA, but relatively sparse in the STLD (Figure 2C). To specifically label this neuronal population, a triple-virus intersectional strategy, a method established for defining neurons based on their convergent input and output connectivity [53,54], was employed: rAAV 2/1-Cre was injected into the PFC, a retrograde AAV expressing FLP recombinase was delivered into the BNST, and a dual-recombinase-dependent mCherry-expressing AAV was introduced into the BLA. This approach enabled specific labeling of BLA neurons that receive input from the PFC and project to the BNST (Figure 2D).

Next, to determine whether the direct and indirect pathways converge onto common BNST neurons, we employed an intersectional dual-virus approach. To label BNST neurons potentially engaged by the indirect PFC → BLA → BNST pathway, we used a trans-synaptic strategy: injecting rAAV 2/1-Cre into PFC, Cre-dependent WGA-FLP AAV into BLA, and FLP-dependent EGFP AAV into BNST. This approach labels BNST neurons receiving BLA input (“PFC → BLA → BNST” neurons). In the same animals, we labeled BNST neurons receiving direct PFC input by injecting rAAV 2/1-Cre into the PFC and a Cre-dependent mCherry AAV into BNST (“PFC → BNST” neurons).

Strikingly, this intersectional viral mapping revealed that the EGFP-positive (potential PFC → BLA → BNST) and mCherry-positive (PFC → BNST) neuronal populations within the BNST were largely distinct and spatially segregated from each other (Figure 2E). This clear anatomical segregation demonstrates that the direct and potential indirect pathways from the PFC are channeled through different cellular substrates within the BNST, establishing the fundamental architecture of parallel processing in this affective circuit (Figure 2F).

The parallel circuit architecture revealed in Figure 2 suggests that the PFC → BLA → BNST and PFC → BNST pathways may serve distinct functions. An intriguing hypothesis is that BLA relay neurons integrate information to coordinate a broad range of behavioral outputs. To directly test this, we examined the effects of specifically manipulating the BLA relay neurons within the PFC → BLA → BNST pathway on a series of behavioral deficits.

### 3.3. BLA Relay Neurons in the PFC → BLA → BNST Pathway Integrate Affective and Social Behaviors

**Figure 3:**
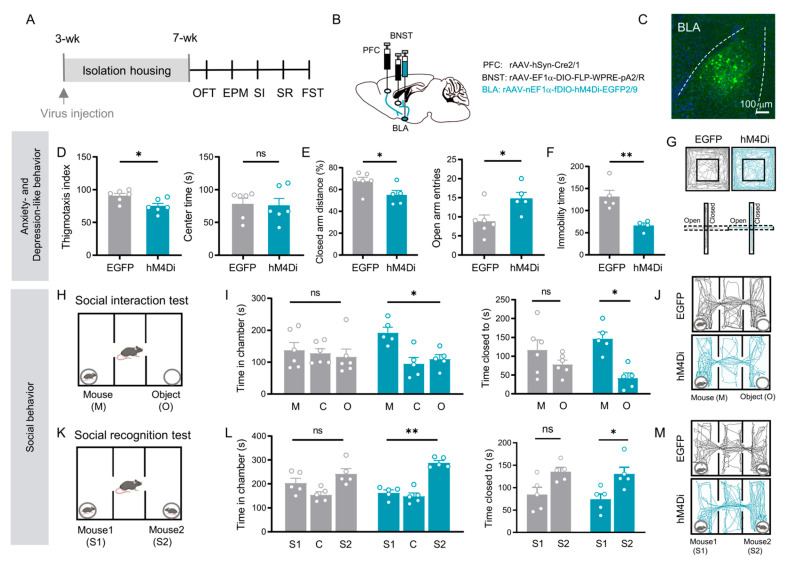
Chemogenetic inhibition of BLA relay neurons in the PFC → BLA → BNST pathway rescues both affective and social deficits induced by social isolation. (**A**) Experimental timeline. (**B**) Schematic of viral injections: rAAV 2/1-Cre into PFC; Retro-Ef1α-DIO-FLP into BNST; and nEf1α-fDIO-hM4Di (inhibition) or EGFP (control) into BLA. (**C**) Representative image of labeled PFC → BLA → BNST relay neurons. Scale bar: 100 µm. (**D**) Open field test: EGFP (*n* = 6) vs. hM4Di (*n* = 6) thigmotaxis index (*p* = 0.021, Student’s *t* test) and center time (*p* = 1.000, Mann–Whitney U test). (**E**) Elevated plus maze: EGFP (*n* = 6) vs. hM4Di (*n* = 5) distance in closed arms (*p* = 0.042, Student’s *t* test) and open arm entries (*p* = 0.030, Mann–Whitney U test). (**F**) Forced swim test: immobility time in EGFP (*n* = 5) vs. hM4Di (*n* = 4) groups (*p* = 0.006, Student’s *t* test). (**G**) Representative OFT (**upper**) and EPM (**lower**) trajectories for EGFP (**left**) and hM4Di (**right**) groups. (**H**) Social interaction test schematic. (**I**) Social interaction results: EGFP (*n* = 6) vs. hM4Di (*n* = 5) chamber time (mouse vs. object: EGFP *p* = 0.638, hM4Di *p* = 0.025) and proximity time (mouse vs. object: EGFP *p* = 0.189, hM4Di *p* = 0.017; paired *t* test). (**J**) Representative SI trajectories for EGFP (**upper**) and hM4Di (**lower**). (**K**) Social recognition test schematic. (**L**) Social recognition results: EGFP (*n* = 5) vs. hM4Di (*n* = 5) chamber time (S1 vs. S2: EGFP *p* = 0.386, hM4Di *p* = 0.001) and proximity time (S1 vs. S2: EGFP *p* = 0.073, hM4Di *p* = 0.018; paired *t* test). (**M**) Representative SR trajectories for EGFP (**upper**) and hM4Di (**lower**). All data: mean ± SEM; * *p* < 0.05, ** *p* < 0.01; ns = not significant.

For cell-type-specific manipulation of the BLA relay neurons within the PFC → BLA → BNST pathway, an intersectional viral strategy was implemented. A rAAV 2/1-Cre was injected into the PFC, and a retrograde AAV expressing FLP recombinase and redbeads were delivered into the BNST (injection site in BNST was shown in Figure S1B). In the BLA, a dual-recombinase-dependent AAV expressing either the inhibitory designer receptor hM4D(Gi) or EGFP was introduced (Figure 3B). This approach confined transgene expression to BLA neurons that receive input from the PFC and project to the BNST, thereby selectively labeling and enabling functional manipulation of the specific PFC → BLA → BNST relay neurons (Figure 3B,C). The viral expression and injection sites for each mouse in behavioral experiments were histologically verified, with their brain section reconstruction diagrams provided in the Supplementary Materials (Figure S1D).

Chemogenetic inhibition of these defined BLA relay neurons (via CNO administration) in socially isolated mice produced a broad rescue of behavioral deficits. Treated mice showed a significant improvement in anxiety- and depressive-like behaviors, evidenced by reduced thigmotaxis index in the open field (*p* = 0.021; Figure 3D), decreased the percentage of the closed arm distance, increased open arm entries in the elevated plus maze (*p* < 0.05; Figure 3E), and decreased immobility time in the forced swim test (*p* = 0.006; Figure 3F).

Critically, the social deficits were also rescued. In the sociability phase, mice with inhibited BLA relay neurons spent significantly more time in the chamber with a stranger mouse (time spent in mouse chamber vs. in object chamber: EGFP: *p* = 0.638; hM4Di: *p* = 0.025, Figure 3I left) and in direct proximity to it (time spent closed to mice vs. closed to object: EGFP: *p* = 0.189; hM4Di: *p* = 0.017, Figure 3I right), compared to the object chamber. During the social novelty phase, these mice successfully discriminated the novel mouse, spending more time in its chamber (time spent in novel mice chamber vs. in familiar mice chamber: EGFP: *p* = 0.386; hM4Di: *p* = 0.001, Figure 3L left) and in proximity to it (time spent closed to novel mice vs. closed to familiar mice: EGFP: *p* = 0.010; hM4Di: *p* = 0.018, Figure 3L right) over the familiar mouse.

These results demonstrate that chemogenetic inhibition of the specific BLA relay neurons within the PFC → BLA → BNST pathway is sufficient to broadly improve the anxiety-depressive-like and social deficits induced by social isolation.

### 3.4. BNST Neurons in the PFC → BLA → BNST Pathway Selectively Mediate Affective Deficits

**Figure 4:**
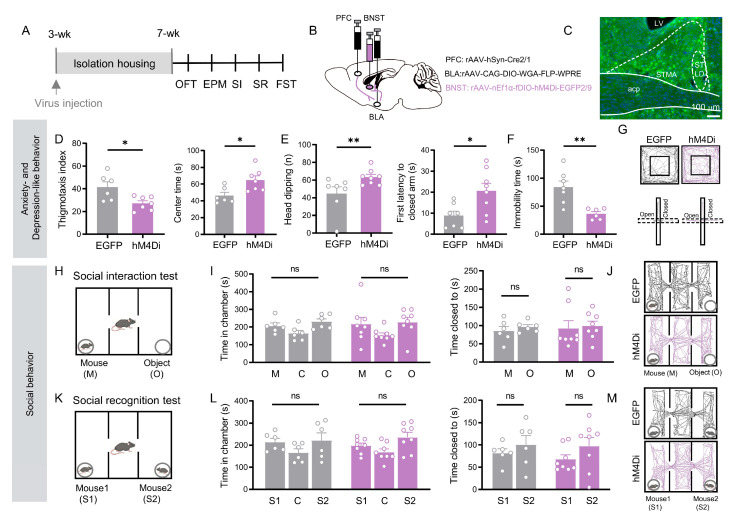
Chemogenetic inhibition of BNST neurons receiving indirect PFC inputs relayed through the BLA alleviate social isolation-induced anxiety-like and depression-like behaviors, but not deficits in social motivation or recognition. (**A**) Experimental timeline. (**B**) Schematic of viral injections: rAAV 2/1-Cre into PFC; CAG-DIO-WGA-FLP into BLA; and nEf1α-fDIO-hM4Di (inhibition) or EGFP (control) into BNST. (**C**) Representative image of labeled BNST neurons in the PFC → BLA → BNST circuit. Scale bar: 100 µm. (**D**) Open field test: hM4Di group (*n* = 7) showed reduced thigmotaxis (*p* = 0.029, Welch’s *t* test) and increased center time (*p* = 0.016, Student’s *t* test) versus EGFP controls (*n* = 6). (**E**) Elevated plus maze: hM4Di group (*n* = 8) increased head-dipping (*p* = 0.035) and closed-arm latency (*p* = 0.019) versus EGFP (*n* = 7). (**F**) Forced swim test: hM4Di group (*n* = 6) showed reduced immobility (*p* = 0.003) versus EGFP (*n* = 7). ((**E**,**F**), Student’s *t* test) (**G**) Representative OFT/EPM trajectories. (**H**–**M**) Social behavior tests: no significant differences were observed between groups in social interaction (**I**,**J**) or social recognition (**L**,**M**) (Figure (**I**,**L**), all *p* > 0.05, paired *t* test). All data: mean ± SEM; * *p* < 0.05, ** *p* < 0.01; ns = not significant.

To clarify the role of BNST neurons in the PFC → BLA → BNST pathway, we next examined this specific population. Using the same intersectional viral targeting approach (Figure 4B), chemogenetic inhibition in socially isolated mice selectively improved their affective deficits. Treated mice showed reduced anxiety-like behavior. In the open field test, their thigmotaxis index increased and center time lengthened (*p* < 0.05, Figure 4D). In the elevated plus maze, risk-assessment head-dipping decreased and entry into the closed arms was delayed (*p* < 0.05; Figure 4E). Immobility time in the forced swim test was also significantly shorter (*p* = 0.003; Figure 4F).

Notably, however, the same manipulation left social behaviors unchanged. Inhibited mice showed no preference for a stranger mouse over an object, nor for a novel over a familiar mouse (*p* > 0.05; Figure 4I,L). Thus, inhibition of the BNST neurons in the PFC → BLA → BNST pathway specifically reverses anxiety- and depressive-like behaviors, yet has no effect on social motivation or social recognition.

The distinct behavioral profiles resulting from manipulation of the PFC → BLA → BNST pathway prompted us to investigate the role of the PFC → BNST projection in social behavior. We hypothesized that this direct pathway might specifically contribute to social deficits induced by isolation.

### 3.5. BNST Neurons in the PFC → BNST Pathway Specifically Mediate Social Recognition

**Figure 5:**
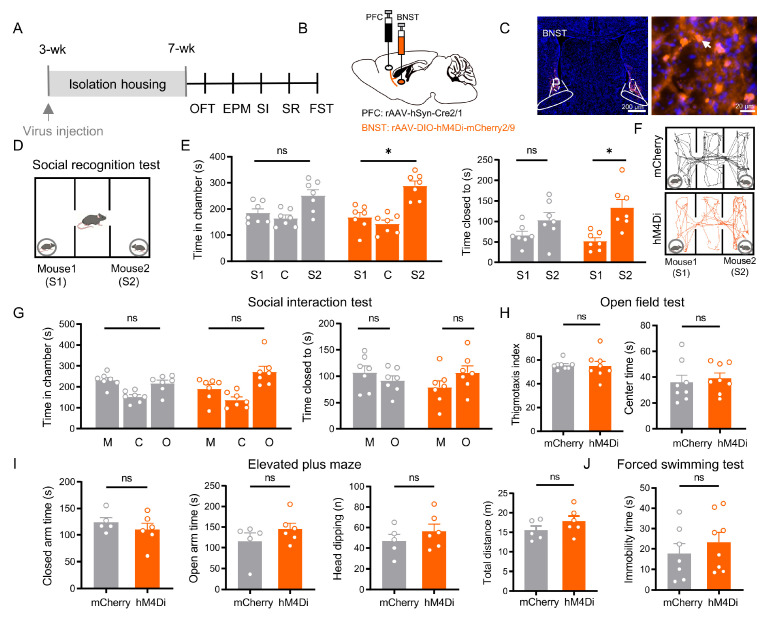
BNST neurons receiving direct PFC inputs are specifically required for social recognition, independent of affective state and social motivation. (**A**) Experimental timeline. (**B**) Schematic of viral injections: rAAV 2/1-Cre into PFC and Ef1α-DIO-mCherry (control) or Ef1α-DIO-hM4Di (inhibition) into BNST. (**C**) Representative images of labeled PFC-innervated BNST neurons (Scale bar: 200 µm) and a magnified image (**right**, Scale bar: 20 µm). Arrows indicate BNST neurons that innervated by the PFC. (**D**) Social recognition test schematic. (**E**) Social recognition results: while control (mCherry, *n =* 7) mice showed no preference, chemogenetic inhibition (hM4Di, *n =* 7) significantly impaired the discrimination between familiar (S1) and novel (S2) mice in both chamber time (*p* = 0.011) and proximity time (*p* = 0.020, paired *t* test). (**F**) Representative SR trajectories for mCherry (**upper**) and hM4Di (**lower**). (**G**) Social interaction results: both groups showed no significant preference between mouse and object (all *p* > 0.05, paired *t* test). (**H**) Open field test: no significant differences were observed in anxiety-related measures (thigmotaxis index, *p* = 0.574; center time, *p* = 0.646). (**I**) Elevated plus maze: no significant differences were observed (closed arm time, *p* = 0.392; open arm time, *p* = 0.429; head-dipping, *p* = 0.338, total distance, *p* = 0.230). (**J**) Forced swim test: immobility time showed no significant difference between groups (*p* = 0.438). All data: mean ± SEM; * *p* < 0.05; ns = not significant (*p* > 0.05).

We targeted BNST neurons receiving direct input from the PFC (PFC injection site shown in Figure S2A) by injecting a rAAV 2/1-Cre into the PFC and a Cre-dependent hM4Di AAV into the BNST. Chemogenetic inhibition of these direct pathway neurons in socially isolated mice selectively improved social recognition memory. Treated mice showed a significant preference for a novel mouse over a familiar one (time spent closed to novel mice vs. closed to familiar mice: mCherry: *p* = 0.119; hM4Di: *p* = 0.011, Figure 5E left), spending more time in the novel mouse’s chamber than in the familiar mouse’s chamber (mCherry: *p* = 0.142; hM4Di: *p* = 0.02; Figure 5E right). Viral expression and histological verification of injection sites for all mice in this experiment are provided in the Supplementary Materials (Figure S2B).

In contrast, this manipulation produced no significant effects on other behavioral domains: social motivation remained impaired, with no preference for a stranger mouse over an object (*p* > 0.05; Figure 5G); anxiety-like behaviors were unchanged across all measured parameters in the OFT and EPM (all *p* > 0.05; Figure 5H,I); and depressive-like behavior was unaffected, showing no alteration in FST immobility (*p* > 0.05; Figure 5J).

## 4. Discussion

Chronic social isolation stress induces a complex array of behavioral abnormalities, including affective disorders and social cognitive deficits. By systematically mapping and dissecting prefrontal efferent pathways, we reveal a hierarchical circuit mechanism underlying stress-induced behavioral deficits. Our findings identify the BLA as a critical relay that amplifies stress signals through the indirect PFC → BLA → BNST pathway. Within the BNST, two distinct neuronal ensembles—defined by their differential inputs (indirect via BLA vs. direct from PFC)—differentially regulate affective and social behaviors.

### 4.1. BLA: A Hub for Integrating and Amplifying Stress Signals

In socially isolated mice, inhibition of BLA neurons within the PFC → BLA → BNST pathway significantly alleviated anxiety-like behaviors, depression-like behaviors, and improved social motivation and recognition. This result not only further solidifies the role of the BLA as a key hub in stress information processing—consistent with its fundamental function in emotional valence assessment [23]—but also suggests a specific role for this pathway in integrating and transmitting chronic social stress signals.

Our findings indicate that the BLA neurons at this relay node function as an integrative hub with divergent outputs. The simultaneous rescue of both affective and social behaviors upon their inhibition strongly suggests that these neurons broadcast the integrated stress signals to multiple downstream targets. Specifically, their projections to BNST mediate the affective phenotypes, while their outputs to other regions (potentially including but not limited to the central amygdala, or other cortical areas) are likely critical for the observed social motivation and recognition deficits.

Our findings regarding anxiety and depression align with the established model that the PFC → BLA pathway is a critical node in chronic stress pathophysiology. Studies show that stimulating PFC-to-BLA projections can induce negative affective states [55]. Moreover, chronic stress paradigms, such as restraint stress, selectively enhance glutamatergic transmission from the PFC to the BLA, creating a persistent excitation/inhibition imbalance that is a key upstream event in anxiety development [56]. Extending this to a psychosocial stress model, we provide direct functional evidence: consistent with findings in other stress models [57], we observed a significant increase in BLA neuronal activation (c-Fos expression) following chronic social isolation. This supports the hypothesis that sustained, aberrant drive from the PFC underlies BLA hyperactivity. Collectively, this suggests social isolation, as a potent psychosocial stressor, engages similar synaptic and molecular mechanisms to pathologically strengthen PFC input to the BLA.

A key advancement of our study is demonstrating that this potentiated input converges on a specific subset of BLA neurons that project to the BNST, acting as a critical relay bottleneck. The BNST is essential for sustaining anxiety and negative affect, and BLA → BNST projections are strongly implicated in anxiety-like behaviors [39,40,41,52]. Our targeted inhibition at this convergent node blocked the propagation of amplified stress signals to this central effector region for negative emotion, thereby reversing the anxiety and despair phenotypes. However, the complete rescue of social deficits upon BLA relay neuron inhibition highlights that these neurons also influence social behavior through projections independent of the BNST.

Regarding social behavior, inhibition of these pathway-defined BLA relay neurons rescued the co-occurring deficits in social approach motivation and social novelty recognition caused by isolation. The BLA is a pivotal hub for integrating social contextual information from associational cortices and is crucial for social recognition memory [58]. Chronic stress is a known inducer of BLA hyperexcitability [59], and such dysregulated BLA activity is a major driver of behavioral pathology across stress models [60]. We propose that this pathological hyperactivity likely impairs the fidelity of social signal processing within the BLA. Specifically, it may degrade the network’s ability to accurately encode and discriminate fine-grained social cues (e.g., individual identity) necessary for normal social recognition, directly resulting in the observed deficit.

### 4.2. BNST: A Parallel Processing Center for Affective and Social Information

The study identified a significant functional dissociation within the BNST: chemogenetic inhibition of BNST neurons postsynaptic to the PFC → BLA → BNST pathway specifically alleviated affective symptoms, whereas chemogenetic inhibition of BNST neurons in the direct PFC → BNST pathway selectively rescued social recognition deficits. This double dissociation provides compelling evidence for the BNST as a center for parallel information processing [32,61].

#### 4.2.1. BNST Neurons in the PFC → BLA → BNST Pathway Mediate Affective Behaviors

The study demonstrates that manipulating BNST neurons within the PFC → BLA → BNST pathway specifically modulates affective behaviors. This finding aligns with the established role of the BLA → BNST pathway in anxiety. Anatomical studies show that the BLA provides dense glutamatergic inputs to specific BNST subregions [62], and direct manipulation of this pathway bidirectionally regulates anxiety-like behavior [63]. Our intervention effectively blocked the BLA-driven “anxiety stream.” These BNST neurons largely comprise CRH-expressing GABAergic cell populations [36,37], which coordinate an anxiety state through divergent downstream targets (e.g., lateral hypothalamus LH and ventral tegmental area VTA) [41]. Furthermore, the BNST contains intrinsically antagonistic microcircuits [64], and neuromodulators like serotonin also regulate the activity of these circuits via specific receptors [37].

#### 4.2.2. BNST Neurons in the Direct PFC → BNST Pathway Independently Regulates Social Recognition

Parallel and functionally distinct, manipulating BNST neurons in the direct PFC → BNST pathway specifically affected social recognition memory. This suggests that PFC control over social cognition can occur directly, bypassing emotional processing by the BLA. Vasopressin (AVP)-expressing neurons in the BNST are crucial for social investigation and recognition [44,65], while the oxytocin (OT) system in the anteromedial BNST plays a key role in stress-induced social avoidance [66,67]. These social information processing systems operate independently of the BLA-driven emotional pathway, highlighting the functional specialization of BNST microcircuitry.

### 4.3. Limitations and Conceptual Framework

Our study has several limitations that define the scope of our findings. The phenomenological nature of our approach—relying primarily on chemogenetic inhibition without complementary electrophysiological or imaging methodologies—means we cannot characterize the dynamic properties of these circuits or their information-coding schemes. We have not performed electrophysiological or immunohistochemical analyses of pathway-specific neurons, nor have we recorded their activity in freely behaving animals. While such studies fall beyond the scope of the current work, they would further clarify the cellular and dynamic properties of these circuits and represent an important direction for future research. Additionally, while we identify necessary pathways, the synaptic and molecular adaptations underlying social isolation-induced circuit dysregulation remain uncharacterized. Finally, it should be noted that all experiments were conducted in adult male mice. Whether the circuit organization and behavioral functions identified here generalize to females remains an open question, and future studies are needed to systematically examine potential sex differences in these pathways.

Nevertheless, our functional mapping provides a crucial anatomical framework for understanding how chronic stress produces coordinated yet dissociable behavioral symptoms. The described hierarchical organization—with dysregulated PFC output driving BLA hyperactivity that subsequently engages separable BNST microcircuits—offers a parsimonious explanation for the frequent comorbidity of affective and social symptoms in stress-related disorders [7,33]. This model emphasizes that complex behavioral syndromes can emerge from the differential recruitment of parallel, functionally specialized pathways downstream of a common hub, revealing new anatomical targets for developing symptom-specific interventions for stress-related psychopathology [25,68]—for instance, targeting BNST microcircuits receiving BLA input (e.g., CRH neurons) to alleviate anxiety, or modulating the direct PFC-input BNST pathway (e.g., AVP/OT neurons) to improve social function [68,69]. These findings are echoed in human studies, such as enhanced BNST connectivity in panic disorder [9] and aberrant BNST activity in obsessive-compulsive disorder [70], suggesting that dysregulation of the BLA-BNST circuit is a cross-species conserved, core mechanism mediating stress-related psychopathology [43,71].

## 5. Conclusions

In conclusion, our study reveals that within the BNST, two functionally distinct neuronal ensembles—defined by their differential input sources—serve as parallel processing modules for stress-induced behavioral deficits. We identify a functional hierarchy wherein the BLA acts as a critical stress amplifier within an indirect PFC → BLA → BNST pathway, ultimately driving the ensemble that governs affective symptoms. Conversely, the ensemble receiving direct PFC input independently regulates social recognition deficits. This functional dissociation establishes a novel circuit model in which separable BNST microcircuits process emotional and social information in parallel. By delineating this hierarchical organization and pinpointing dissociable BNST populations, our work provides a concrete anatomical and functional basis for developing precise, symptom dimension-specific interventions for complex stress-related psychopathologies.

## Figures and Tables

**Table 1:** Viral Vectors Used for Anatomical and Functional Labeling.

Purpose	Brain Area		
	PFC	BLA	BNST
label the PFC → BLA → BNST relay neurons	rAAV 2/1-Cre	rAAV 2/9-nEfIα-fDIO-mCherry-WPRE pA	rAAV 2/R-EfIα-DIO-FLP-WPRE pA
label the BNST neurons in the PFC → BLA → BNST circuit	rAAV 2/1-Cre	rAAV 2/9-CAG-DIO-WGA-FLP-WPRE-hGH pA	rAAV 2/9-nEfIα-fDIO-EGFP-WPRE-hGH pA
chemogenetic inhibits the PFC → BLA → BNST relay neurons	rAAV 2/1-Cre	rAAV 2/9-nEfIα-fDIO-hM4Di-EGFP-WPRE-hGH pA	rAAV 2/R-EfIα-DIO-FLP-WPRE pA
chemogenetic inhibits the BNST neurons in the PFC → BLA → BNST circuit	rAAV 2/1-Cre	rAAV 2/9-CAG-DIO-WGA-FLP-WPRE-hGH pA	rAAV 2/9-nEfIα-fDIO-hM4Di-EGFP-WPRE-hGH pA
chemogenetic inhibits the PFC-innervated BNST neurons	rAAV 2/1-Cre	/	rAAV 2/9-EfIα-DIO-hM4Di-mCherry

## Data Availability

Data presented in the article are available upon reasonable request.

## Supplementary Materials

The following supporting information can be downloaded at: https://www.mdpi.com/article/10.3390/cells15020116/s1, Figure S1: Viral Injection and Expression Verification for Chemogenetic Inhibition of the BLA Relay Neurons Within the PFC → BLA → BNST Pathway; Figure S2: Viral Targeting and Expression for Chemogenetic Inhibition of the BNST Neurons Innervated by the PFC.

## Abbreviations

The following abbreviations are used in this manuscript:

PFC	Prefrontal cortex
BLA	basolateral amygdala
BNST	bed nucleus of the stria terminalis
STMA	bed nucleus of the stria terminalis, medial division, anterior part
STLD	bed nucleus of the stria terminalis, lateral division, dorsal part
GH	Group-housing
IH	Isolation-housing
OFT	Open field test
EPM	Elevated plus maze
SI	Social interaction test
SR	Social recognition test
TS	Tail suspension
FST	Forced swimming test

## Appendix A

**Table A1:** Chemicals and antibody.

Reagent	Source	Identifier	Concentration/Volume	Storage Temperature
**Antibodies**				
Anti-cFos polyclonal antibody	Abcam, Cambridge, Cambridgeshire, UK	ab190289	1:1000	−20 °C
Alexa Flour 488 donkey anti-rabbit IgG (H + L)	Invitrogen, Carlsbad, CA, USA	A21206	1:500	4 °C
**Virus**				
rAAV2/1-hSyn-Cre-WPRE-hGH pA	Brain VTA, Wuhan, Hubei, China	PT-0136	200 nL	−80 °C
rAAV2/9-CAG-DIO-WGA-FLP-WPRE-hGH pA	Brain VTA, Wuhan, Hubei, China	PT-0557	200 nL	−80 °C
rAAV2/9-nEf1α-fDIO-hM4Di-EGFP-WPRE-hGH pA	Brain VTA, Wuhan, Hubei, China	PT-0159	200 nL	−80 °C
rAAV2/R-Ef1α-DIO-FLP-WPRE pA	Brain VTA, Wuhan, Hubei, China	PT-0075	200 nL	−80 °C
rAAV2/9-nEf1α-fDIO-mcherry-WPRE pA	Brain VTA, Wuhan, Hubei, China	PT-0339	200 nL	−80 °C
rAAV2/9-hSyn-DIO-hM4Di-mcherry	OBiO, Shanghai, China	H2481	200 nL	−80 °C
rAAV2/9-hSyn-EGFP-WPRE-hGH polyA	Brain VTA, Wuhan, Hubei, China	PT-0241	200 nL	−80 °C
pAAV2/R-hSyn-MCS-mCherry-3FLAG	OBiO, Shanghai, China	AOV063	200 nL	−80 °C
pAAV2/R-hSyn-MCS-EGFP-3FLAG	OBiO, Shanghai, China	AOV062	200 nL	−80 °C
**Chemical reagent**				
Clozapine-N-oxide	MedChemExpress, Monmouth Junction, NJ, USA	HY-17366	1 mg/kg 0.1 μg	−20 °C
