# Supplementary material for: Separate BNST Microcircuits Targeted by Direct Versus Amygdala-Relayed Prefrontal Inputs Mediate Dissociable Phenotypes After Isolation
Source: Cells. 2026 Jan 8;15(2):116. doi: 10.3390/cells15020116 (PMC12839825; doi:10.3390/cells15020116)
Supplement: Supplementary file 1 [file cells-15-00116-s001.zip › Supplementary Figure Legends.pdf]

Figure S1: Viral Injection and Expression Verification for Chemogenetic Inhibition of the BLA Relay Neurons Within the PFC→BLA→BNST Pathway

(A) Schematic diagram of the viral injection strategy for chemogenetic inhibition of the BLA relay neurons within the PFC→BLA→BNST pathway.

(B) Representative image showing the expression of retrograde tracer (red beads) in the BNST, confirming the injection site. Scale bar: 100 μm. Blue: DAPI.

(C) Representative images demonstrating the expression of the BLA relay neurons within the PFC→BLA→BNST pathway. Scale bar: 100 μm. Blue: DAPI.

(D) Reconstructed injection sites and expression maps for mice included in the study, depicting targeting accuracy within the BNST (top) and BLA (bottom). Each symbol represents a single animal.

Figure S2: Viral Targeting and Expression for Chemogenetic Inhibition of the BNST Neurons Innervated by the PFC.

(A) Viral injection strategy (left) and the injection site in PFC (right). (B) Reconstructed histological verification of injection and expression sites in the BNST (top) and PFC (bottom). The spread of the control virus (rAAV-Ef1α-DIO-mCherry) and the inhibitory chemogenetic virus (rAAV-Ef1α-DIO-hM4Di-mCherry) is shown for animals included in the study. Each symbol represents a single animal.
