# Supplementary figures and images for: Separate BNST Microcircuits Targeted by Direct Versus Amygdala-Relayed Prefrontal Inputs Mediate Dissociable Phenotypes After Isolation
Source: Cells. 2026 Jan 8;15(2):116. doi: 10.3390/cells15020116 (PMC12839825; doi:10.3390/cells15020116)

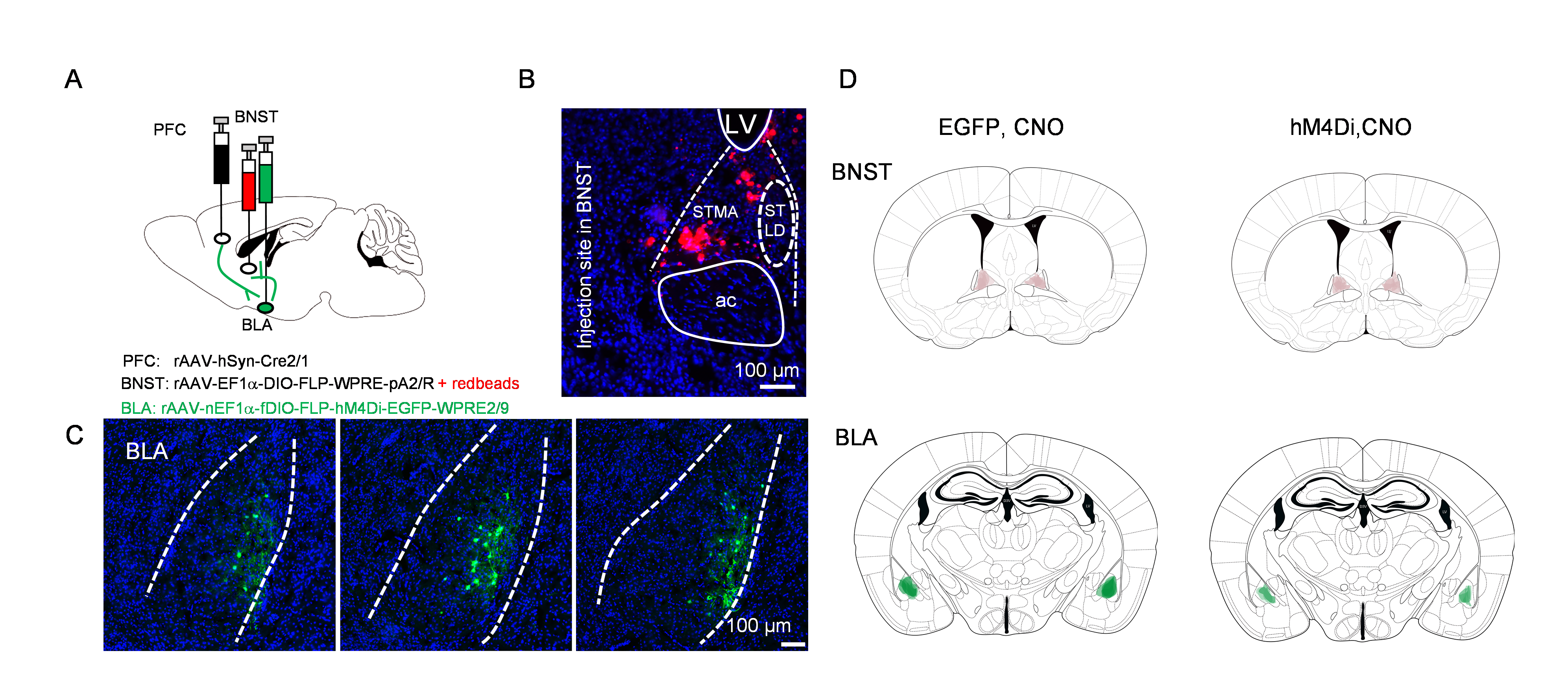

Supplement: Supplementary file 1 [file cells-15-00116-s001.zip › Figure S1.png]

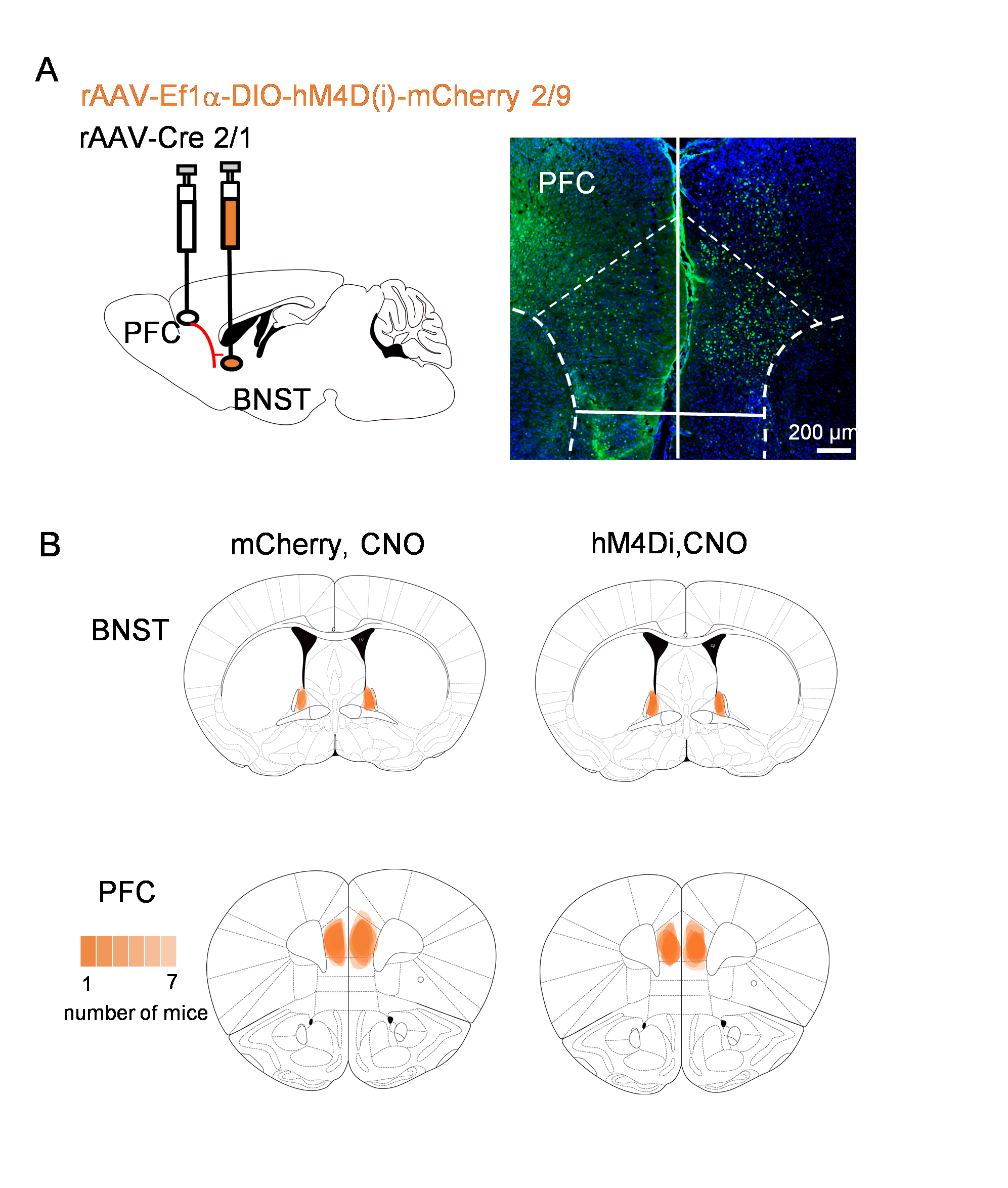

Supplement: Supplementary file 1 [file cells-15-00116-s001.zip › Figure S2.png]
